# Supplementary material for: Predictive pursuit emerges in high-dimensional recurrent neural networks
Source: bioRxiv. 2026 Apr 27:2026.04.23.720457. Preprint. [Version 1] doi: 10.64898/2026.04.23.720457 (PMC13142312; doi:10.64898/2026.04.23.720457)
Supplement: Supplement 1 [file NIHPP2026.04.23.720457v1-supplement-1.pdf]

bioRxiv preprint doi: https://doi.org/10.64898/2026.04.23.720457; this version posted April 27, 2026. The copyright holder for this preprint (which was not certified by peer review) is the author/funder, who has granted bioRxiv a license to display the preprint in perpetuity. It is made available under aCC-BY 4.0 International license.

A Task and the RNN models

A.1 RNNs as pursuit agents

We consider the setting where two agents ("RNN-agent" and "target-agent") traverse a square environment through finite length paths. The target-agent starts at position $\mathbf{z}_{\text{target}}(0) = \big(x_{\text{target}}(0),\, y_{\text{target}}(0)\big)^{\intercal} \in [-L/2, L/2] \times [-L/2, L/2]$ and subsequently moves to positions $\mathbf{z}_{\text{target}}(0) \to \mathbf{z}_{\text{target}}(1) \to ... \to \mathbf{z}_{\text{target}}(T)$, where the path is sampled using a model of motion that is biased towards straight paths and avoidance of walls [19, 27] (Fig. 1A). This path can alternatively be defined by $\mathbf{z}_{\text{target}}(0)$ and a sequence of movement directions, $\theta_{\text{target}}(t) \in [-\pi, \pi]$, and speeds, $v_{\text{target}}(t) \in [0, \infty)$, such that

$$\begin{aligned}\mathbf{z}_{\text{target}}(t+1) &= \mathbf{z}_{\text{target}}(t) + v_{\text{target}}(t) \cdot \Delta t \cdot \big(\cos\theta_{\text{target}}(t),\, \sin\theta_{\text{target}}(t)\big)^{\intercal} \\ &= \mathbf{z}_{\text{target}}(t) + \Delta t \cdot \big(v^x_{\text{target}}(t),\, v^y_{\text{target}}(t)\big)^{\intercal}\end{aligned} \tag{1}$$

for small $\Delta t \in \mathbb{R}^+$ and $t = 1, ..., T$, and where $v^x_{\text{target}}(t) = v_{\text{target}}(t)\cos\theta_{\text{target}}(t)$ and $v^y_{\text{target}}(t) = v_{\text{target}}(t)\sin\theta_{\text{target}}(t)$. The RNN-agent similarly starts at a random position in the environment, $\mathbf{z}_{\text{RNN}}(0) = \big(x_{\text{RNN}}(0),\, y_{\text{RNN}}(0)\big)^{\intercal} \in [-L/2, L/2] \times [-L/2, L/2]$ (Fig. 1A). We train the RNN to generate a sequence of movement directions, $\theta_{\text{RNN}}(t) \in [-\pi, \pi]$, and speeds, $v_{\text{RNN}}(t) \in [0, v_{\max})$, such that the trajectory of the RNN-agent, $\mathbf{z}_{\text{RNN}}(0) \to \mathbf{z}_{\text{RNN}}(1) \to ... \to \mathbf{z}_{\text{RNN}}(T)$, is as close to the target's end position as possible (Fig. 1C). This is enforced by the loss function,

$$\mathcal{L}_{\text{end}} = ||\mathbf{z}_{\text{RNN}}(T) - \mathbf{z}_{\text{target}}(T)||_2. \tag{2}$$

To learn an RNN model that is able to perform pursuit, we utilize the following architecture (Fig. 1B). Inputs, in the form of $\mathbf{u}(t) = \big(v^x_{\text{target}}(t), v^y_{\text{target}}(t)\big)^{\intercal}$, are projected into the recurrent layer of an RNN via weights $\mathbf{W}^{\text{in}} \in \mathbb{R}^{N\times 2}$, where N is the number of hidden units. The recurrent units process the input via the weights $\mathbf{W}^{\text{rec}} \in \mathbb{R}^{N\times N}$, and drive activations in output units via the weights $\mathbf{W}^{\text{out}} \in \mathbb{R}^{2\times N}$. More explicitly, the dynamics of the RNN model are given by

$$\mathbf{r}(t+1) = \sigma\left[\mathbf{W}^{\text{rec}}\mathbf{r}(t) + \mathbf{W}^{\text{in}}\mathbf{u}(t)\right], \tag{3}$$

$$\big(\theta_{\text{RNN}}(t+1),\, v_{\text{RNN}}(t+1)\big)^{\intercal} = \mathbf{W}^{\text{out}}\mathbf{r}(t+1) \tag{4}$$

where $\mathbf{r}(t) \in \mathbb{R}^N$ are the activations of the recurrent units at time t, $\sigma(\cdot)$ is the activation function, and $\theta_{\text{RNN}}(t)$ and $v_{\text{RNN}}(t+1)$ are the generated movement direction and speed for the RNN-agent at time t. To prevent the RNN from using unreasonably large instantaneous speeds to perform pursuit, we threshold the outputted $v_{\text{RNN}}(t+1)$, setting $v_{\text{RNN}}(t+1) = \min\{v_{\text{RNN}}(t+1), v_{\max})\}$, where $v_{\max} \in \mathbb{R}^+$. Given the output $\theta_{\text{RNN}}(t)$, $v_{\text{RNN}}(t)$, we can generate the trajectory of the RNN-agent, $\mathbf{z}_{\text{RNN}}(0) \to \mathbf{z}_{\text{RNN}}(1) \to ... \to \mathbf{z}_{\text{RNN}}(T)$ by using $\theta_{\text{RNN}}(t+1)$ and $v_{\text{RNN}}(t+1)$ to get $v^x_{\text{RNN}}(t+1)$ and $v^y_{\text{RNN}}(t)$. Training can then be achieved by using Eq. 2 as the objective function.

In addition to the movement direction and speed of the target agent, in order to successfully achieve pursuit, it is necessary to know where the RNN-agent and target-agent are at the beginning of a trial. Therefore, we additionally learn an embedding of the $\mathbf{z}_{\text{target}}(0)$ and $\mathbf{z}_{\text{RNN}}(0)$ to the initial state of the RNN, via the weights $\mathbf{W}^{\text{back}} \in \mathbb{R}^{N\times 4}$. That is, $\mathbf{r}(0) = \mathbf{W}^{\text{back}}\big(\mathbf{z}_{\text{RNN}}(0), \mathbf{z}_{\text{target}}(0)\big)^{\intercal}$. All parameters associated with the RNN architecture and all hyper-parameters associated with training the RNN are presented in Table S1.

A.2 Low-rank RNNs

To constrain recurrent connectivity, we replace the full recurrent weight matrix with a rank-K factorization. Specifically, instead of learning $\mathbf{W}^{\text{rec}} \in \mathbb{R}^{N\times N}$ freely, we parameterize it as

$$\mathbf{W}^{\text{rec}} = \frac{1}{N}\mathbf{M}\mathbf{N}, \tag{5}$$

where $\mathbf{M} \in \mathbb{R}^{N\times K}$, $\mathbf{N} \in \mathbb{R}^{K\times N}$, and $K \le N$ is the prescribed rank. By construction, $\text{rank}(\mathbf{W}^{\text{rec}}) \le K$. The factor $1/N$ ensures stable scaling of recurrent contributions as N varies. Under this parameterization, the recurrent update becomes

$$\mathbf{r}(t+1) = \sigma\left(\frac{1}{N}\mathbf{M}\mathbf{N}\mathbf{r}(t) + \mathbf{W}^{\text{in}}\mathbf{u}(t) + \mathbf{b}\right), \tag{6}$$

with the same input, output, and loss definitions as in the full-rank case. The recurrent contribution depends only on the projection

$$\kappa(t) = \frac{1}{N}\mathbf{N}\mathbf{r}(t) \in \mathbb{R}^K, \quad \text{and} \quad \mathbf{W}^{\text{rec}}\mathbf{r}(t) = \mathbf{M}\kappa(t). \tag{7}$$

bioRxiv preprint doi: https://doi.org/10.64898/2026.04.23.720457; this version posted April 27, 2026. The copyright holder for this preprint (which was not certified by peer review) is the author/funder, who has granted bioRxiv a license to display the preprint in perpetuity. It is made available under aCC-BY 4.0 International license.

Table S1: **Parameters used to train RNNs on pursuit.** Unless otherwise noted, the parameters presented below were used to train the RNN on the pursuit task.

Parameter	Value
Epochs	100
Batch size	200
Batches per epoch	1000
Path length (T)	50
Δt	0.02
Arena length (L)	1.0 m
Learning rate	2.5^{-5}
N	1024
Activation	ReLU
Weight decay	10^{-4}
Optimizer	Adam
Percent CTs	25%
$v_{\max}$	0.0225

Thus, the recurrent drive lies in the K-dimensional column space of $\mathbf{M}$, constraining network dynamics to evolve within a low-dimensional subspace when $K \ll N$. The number of recurrent parameters is reduced from N^2 to $2NK$.

Parameters were initialized independently as Gaussian random variables:

$$(\mathbf{W}^{\text{in}})_{ij} \sim \mathcal{N}(0, 1), \tag{8}$$

$$\mathbf{M}_{ij} \sim \mathcal{N}\left(0, \frac{\sqrt{N}}{\sqrt{K}}\right), \tag{9}$$

$$\mathbf{N}_{ij} \sim \mathcal{N}(0, 1), \tag{10}$$

so that the recurrent weights are effectively initialized $(\vec{W}^{\text{rec}})_{ij} \sim \mathcal{N}(0, N^{-1})$. For $N, K \to \infty$, this initialization corresponds to the edge of chaos [38, 75], which has been known as a fruitful initialization in the broader deep learning literature [76]. We trained networks across a range of ranks $K \in \{10, 20, 30, 50, 100, 200, \ldots, 1000\}$, with ten independent random initializations per rank and no weight decay. We used a learning rate of 10^{-4}. We trained for $100,000$ epochs, using a single batch per epoch, matching the total training time as in Table S1 but keeping a more frequent record of the loss function values. All other training hyperparameters were held fixed.

B Characteristic trajectories

Inspired by the experimental paradigm developed in Alexander et al. (2022) [7], we train RNNs on trajectories that are sampled from two different families. The first, which are referred to as pseudo-random trials (RTs), are generated using the same approach as has been described elsewhere [19, 27]. The second, which are referred to as characteristic trials (CTs), are generated using the following procedure. First, the target is randomly assigned to one of four starting locations: $\mathbf{z}^{\mathsf{T}}_{\text{target}}(0) = (-L/4, -L/4)$, $(L/4, -L/4)$, $(-L/4, L/4)$, or $(L/4, L/4)$, where L is the length of the square environment and the environment is defined by $[-L/2, L/2] \times [-L/2, L/2]$. Once the starting location of the target-agent is chosen, then the location of the RNN-agent and the starting movement direction of the target-agent are determined. In particular,

$$\mathbf{z}^{\mathsf{T}}_{\text{RNN}}(0) = \begin{cases} (0, L/4) & \text{if } \mathbf{z}^{\mathsf{T}}_{\text{target}}(0) = (-L/4, -L/4) \\ (-L/4, 0) & \text{if } \mathbf{z}^{\mathsf{T}}_{\text{target}}(0) = (L/4, -L/4) \\ (L/4, 0) & \text{if } \mathbf{z}^{\mathsf{T}}_{\text{target}}(0) = (-L/4, L/4) \\ (0, -L/4) & \text{if } \mathbf{z}^{\mathsf{T}}_{\text{target}}(0) = (L/4, L/4) \end{cases} \tag{11}$$

and

$$\theta_{\text{target}}(0) = \begin{cases} 0 & \text{if } \mathbf{z}^{\mathsf{T}}_{\text{target}}(0) = (-L/4, -L/4) \\ \pi & \text{if } \mathbf{z}^{\mathsf{T}}_{\text{target}}(0) = (L/4, -L/4) \\ 0 & \text{if } \mathbf{z}^{\mathsf{T}}_{\text{target}}(0) = (-L/4, L/4) \\ \pi & \text{if } \mathbf{z}^{\mathsf{T}}_{\text{target}}(0) = (L/4, L/4) \end{cases} \tag{12}$$

bioRxiv preprint doi: https://doi.org/10.64898/2026.04.23.720457; this version posted April 27, 2026. The copyright holder for this preprint (which was not certified by peer review) is the author/funder, who has granted bioRxiv a license to display the preprint in perpetuity. It is made available under aCC-BY 4.0 International license.

The target then moves along trajectories that are updated by sampling speeds from the same distribution as the RTs and sampling changes to the head direction from a normal distribution, where the standard deviation in the distribution for CTs is 1 radians/second, as compared to 11.52 radians/second for RTs. Finally, if the target-agent gets sufficiently close to the wall, the movement direction changes by $+\pi/2$ radians if the target-agent is in the upper-right or lower left-hand quadrants, and the movement direction changes by $-\pi/2$ if the target-agent is in the upper-left or bottom-right quadrants. The resulting affect of this turn is to make the target-agent move along the wall, towards $x = 0$. This creates an "L" shaped-like trajectory (Fig. 1F, black lines).

C Egocentric target units

Electrophysiological recordings of rodents performing pursuit have found cells in RSC and PPC that encode the position of the target-agent in the egocentric coordinate system [7, 40]. We asked whether the RNNs developed similar tuning. To investigate this, we followed similar steps to previous work on analyzing egocentric target and border coding [7, 39]. First, we sample 10000 RT trials, computing the distance and angle between the RNN-agent and the target-agent at each time-point. We define this angle as

$$\theta_{\text{ego}} = \arctan \frac{\Delta y}{\Delta x} - \theta_{\text{RNN}}, \tag{13}$$

where θ_{RNN} is the movement direction of the RNN-agent, $\Delta x = x_{\text{target}} - x_{\text{RNN}}$, and $\Delta y = y_{\text{target}} - y_{\text{RNN}}$. Care needs to be used when taking the arctan to ensure the sign of the output is correct.

We similarly save the activations of all units in the RNN at each time-point. We then split the 10000 RT trials into two subsets of 5000 distinct trials and construct the egocentric target ratemaps by binning distance and angle, and averaging the activity associated with time-points that fall within each bin. Because the RNN was trained to pursue the target-agent, there were few time-points with large distance. Following previous analysis of neural data, we set a maximum distance, $d_{\text{max}} = 0.4$ m. Any time-point with distance greater than d_{max} was discarded and not used for analysis. We denote the two constructed egocentric target ratemaps of unit i by $\mathbf{X}^{\text{ego}}_{i,1}, \mathbf{X}^{\text{ego}}_{i,2} \in \mathbb{R}^{r_d \times r_\theta}$, where r_d is the number of bins for distance and r_θ is the number of bins used for angle. We then compute the mean resultant length (MRL) of these egocentric ratemaps [39]. The MRL is given by

$$\text{MRL}(\mathbf{X}^{\text{ego}}_{i,j}) = \left| \frac{1}{r_\theta} \sum_{k=1}^{r_\theta} \frac{1}{r_d} \sum_{l=1}^{r_d} \mathbf{X}^{\text{ego}}_{i,j}(k,l) e^{i\theta_k} \right|, \tag{14}$$

where θ_k is the value corresponding to the center of the kth angular bin (ranging from $-\pi$ to π).

For each unit in the RNN, we have two MRL values, $\text{MRL}(\mathbf{X}^{\text{ego}}_{i,1})$ and $\text{MRL}(\mathbf{X}^{\text{ego}}_{i,2})$, corresponding to the two separate sets of trials. We assess the robustness of the egocentric coding by computing

$$\rho = \frac{\min\{\text{MRL}(\mathbf{X}^{\text{ego}}_{i,1}), \text{MRL}(\mathbf{X}^{\text{ego}}_{i,2})\}}{\max\{\text{MRL}(\mathbf{X}^{\text{ego}}_{i,1}), \text{MRL}(\mathbf{X}^{\text{ego}}_{i,2})\}} \tag{15}$$

A unit is considered robust is this ratio is greater than 0.95. For all units that are robust, we construct the full egocentric target ratemap, $\mathbf{X}^{\text{ego}}_i$, where we use all 10000 trials. The corresponding MRL is $\text{MRL}(\mathbf{X}^{\text{ego}}_i)$.

To assess which robust units have significant egocentric target tuning, we generate 100 shuffled egocentric target ratemaps $\mathbf{X}^{\text{shuff}}_{i,j}$ by permuting the unit activations, with respect to the distances and angles to the target-agent, on each trial. We then consider the distribution of MRL values computed on all shuffled egocentric target ratemaps. We identify units with significant egocentric target tuning as those with $\text{MRL}(\mathbf{X}^{\text{ego}}_i) > 99^{\text{th}}$ percentile of the shuffled distribution (Fig. 2B). Units that are both robust and have significant egocentric target tuning are classified as egocentric target units (ETUs).

C.1 Ablating egocentric target units

Having found units that are classified as ETUs (Fig. 2A, top row), we asked whether they were used by the RNN to perform pursuit. To test this, we perform targeted ablations [27, 28]. This was achieved ordering ETUs by their MRL and ablating the top pN units, where $p \in [0, 1]$ and N is the total number of units in the RNN, by setting $W^{\text{rec}}_{i,e_j} = W^{\text{rec}}_{e_j,i} = 0$, where e_j is the jth ETU and $W^{\text{rec}}_{i,e_j}, W^{\text{rec}}_{e_j,i}$ are the in-going and out-going recurrent weights to unit e_j. Setting these weights to 0 effectively removes unit e_j from the RNN.

To identify the significance of removing ETUs (as compared to other units), we perform two additional ablation experiments. First, we randomly select pN units to ablate. For each p, we perform 25 random selections. This

bioRxiv preprint doi: https://doi.org/10.64898/2026.04.23.720457; this version posted April 27, 2026. The copyright holder for this preprint (which was not certified by peer review) is the author/funder, who has granted bioRxiv a license to display the preprint in perpetuity. It is made available under aCC-BY 4.0 International license.

enables us to assess whether removing ETUs leads to a more significant effect on pursuit performance than randomly removing units. Given that approximately 25% of RNN units were found to be ETUs, this is a strong baseline [28], as randomly selected units will be ETUs with non-trivial probability. Therefore, we also examine how ablating the RNN units with the lowest MRL affects pursuit performance. In this case, we sort all units by their MRL (not just ETUs) and remove the bottom pN units (Fig. 2A, bottom row). This allows us to examine how important egocentric target coding is to pursuit performance.

D Non-predictive control model

To understand the behavior of the RNN model, we compare its performance to a control model that, by definition, is non-predictive. To do this, we consider the locally optimal solution of moving, at each time step, in the direction of where the target-agent is. More precisely, if $\mathbf{z}_{\text{target}}(t)$ is the current position of the target-agent and $\mathbf{z}_{\text{non-pred}}(t)$ is the current position of the non-predictive-agent, then

$$\min_{\theta,v} ||\mathbf{z}_{\text{target}}(t) - \mathbf{z}_{\text{non-pred}}(t)||_2 = ||\mathbf{z}_{\text{target}}(t) - (\mathbf{z}_{\text{non-pred}}(t) + v(t) \cdot \Delta t \cdot [\cos\theta(t), \sin\theta(t)]^\intercal)||_2, \tag{16}$$

is achieved by

$$\theta_{\text{non-pred}}(t) = \arctan\left[\frac{\Delta_y(t)}{\Delta_x(t)}\right], \tag{17}$$

where $[\Delta_x(t),\, \Delta_y(t)]^\intercal = \mathbf{z}_{\text{target}}(t) - \mathbf{z}_{\text{non-pred}}(t)$. Care has to be taken to add π or 2π to Eq. 17, depending on the signs of Δ_x and Δ_y.

With these movement directions, the non-predictive control model generates a trajectory via

$$\mathbf{z}_{\text{non-pred}}(t+1) = \mathbf{z}_{\text{non-pred}}(t) + v_{\text{non-pred}}(t) \cdot \Delta t \cdot \left[\cos\theta_{\text{non-pred}}(t),\, \sin\theta_{\text{non-pred}}(t)\right]^\intercal. \tag{18}$$

Importantly, if $v_{\text{non-pred}}(t)$ is unconstrained, then $\mathbf{z}_{\text{non-pred}}$ can catch-up instantly to $\mathbf{z}_{\text{target}}$. Thus, to ensure a fair comparison, we take $v_{\text{non-pred}}(t)$ to be the same as the velocity of the RNN. That is, $v_{\text{non-pred}}(t) = v_{\text{RNN}}(t)$. This allows us to identify how the RNN differs from the non-predictive control model in terms of its planning ability through trajectory direction it takes.

Note that this is similar to the baseline with which Yoo et al. (2020) [8] compared their non-human primate results with. However, this is a simpler model that captures a fully non-predictive strategy.

D.1 Shortcut metric

To quantify the ability of the RNN to perform anticipatory behavior, we developed the following "shortcut metric". To this end, we measure the average distance between the RNN's trajectory and the trajectory with the minimum path length (i.e., the "globally optimal" solution). The globally optimal trajectory is given by the linear interpolation between the starting position of the RNN-agent, $\mathbf{z}_{\text{RNN}}(0)$, and the end position of the target-agent, $\mathbf{z}_{\text{target}}(T)$,

$$\mathbf{z}_{\text{optimal}}(\alpha) = \alpha \cdot \mathbf{z}_{\text{RNN}}(0) + (1-\alpha) \cdot \mathbf{z}_{\text{target}}(T), \tag{19}$$

where $\alpha \in [0, 1]$ describes the interpolation.

Let $\ell_{\text{RNN}}(t)$ be the minimum distance between the RNN-agent's trajectory at time t and the globally optimal trajectory. That is,

$$\ell_{\text{RNN}}(t) = \min_{\alpha} ||\mathbf{z}_{\text{optimal}}(\alpha) - \mathbf{z}_{\text{RNN}}(t)||_2. \tag{20}$$

We define our shortcut metric, ε_{RNN}, to be the mean distance between the RNN-agent's trajectory and the globally optimal trajectory,

$$\varepsilon_{\text{RNN}} = \langle \ell_{\text{RNN}}(t) \rangle_t, \tag{21}$$

where $\langle \cdot \rangle_t$ is the mean taken over all time steps in the trajectory.

We analogously define $\varepsilon_{\text{non-pred}}$. Comparing the distribution of ε_{RNN} and $\varepsilon_{\text{non-pred}}$ across many sampled trajectories enables us to quantify statistically significant anticipatory behavior.

bioRxiv preprint doi: https://doi.org/10.64898/2026.04.23.720457; this version posted April 27, 2026. The copyright holder for this preprint (which was not certified by peer review) is the author/funder, who has granted bioRxiv a license to display the preprint in perpetuity. It is made available under aCC-BY 4.0 International license.

E Average distance loss

To understand to what extent the predictive behavioral outputs of the RNN model are due to the loss function penalizing the *end* distance between the RNN-agent and the target-agent, we train RNN models using an alternative loss function that penalizes the *average* distance between the RNN-agent and the target-agent. In particular, where as $\mathcal{L}_{\text{end}}$ (Eq. 2) was a function only of $||\mathbf{z}_{\text{RNN}}(T) - \mathbf{z}_{\text{target}}(T)||_2$, the average loss function is given by

$$\mathcal{L}_{\text{average}} = \frac{1}{T}\sum_{t=1}^{T} ||\mathbf{z}_{\text{RNN}}(t) - \mathbf{z}_{\text{target}}(t)||_2. \tag{22}$$

F Periodic boundary condition environment

To test the capacity of RNN models to learn to perform predictive pursuit, we developed an environment with periodic boundary conditions. That is, we connected the left and right walls, as well as the top and bottom walls. This gave rise to the environment having the topology of a torus, with the distance between two points not equal to the Euclidean distance. The RNN model's ability to learn anticipatory behavior on CTs in this environment (Fig. 5A, left), led us to develop a similar environment to test mice in. Below, we discuss each of these environments in detail.

F.1 RNN experiments

To generate the environment with periodic boundaries in simulation, we made the following modifications to our standard environment and process of sampling target-agent trajectories [19, 27]. First, in the standard environment, the target-agent avoids the boundaries by turning away from them as it nears the wall (modeled as an elastic collision). We remove this effect, so that the target-agent in the periodic boundary condition continues towards the wall. And second, on each update of the position of the target-agent, we enforce periodicity of the environment by checking to see if $\mathbf{z}_{\text{target}}(t+1) \in [-L/2, L/2] \times [-L/2, L/2]$. If $\mathbf{z}_{\text{target}}(t+1)$ is not in this domain, then we perform the following correction

$$x_{\text{target}}(t+1) = \begin{cases} L/2 + v_{\text{target}}(t) \cdot \cos\theta_{\text{target}}(t) & \text{if } x_{\text{target}}(t+1) < -L/2 \\ -L/2 + v_{\text{target}}(t) \cdot \cos\theta_{\text{target}}(t) & \text{if } x_{\text{target}}(t+1) > L/2 \end{cases} \tag{23}$$

$$y_{\text{target}}(t+1) = \begin{cases} L/2 + v_{\text{target}}(t) \cdot \sin\theta_{\text{target}}(t) & \text{if } y_{\text{target}}(t+1) < -L/2 \\ -L/2 + v_{\text{target}}(t) \cdot \sin\theta_{\text{target}}(t) & \text{if } y_{\text{target}}(t+1) > L/2 \end{cases} \tag{24}$$

We perform similar corrections to the position of $\mathbf{z}_{\text{RNN}}(t+1)$.

Training RNN models to pursue target-agents in this environment, we find low performance on RTs (median distance to end location of target-agent – RNN = 0.34 m). This is presumably because the target-agent is able to quickly "jump" from one part of the environment to the other, and can even make multiple jumps in a short span of time. This makes it challenging for the RNN-agent to catch the target-agent. However, appropriately defined CTs offer the potential for providing enough structure that the RNN model could potentially learn to predictively pursue the target-agent.

Therefore, we sample CTs in this environment, using the same procedure as was done in the standard environment, with the two modifications listed above. Namely, not implementing the boundary avoidance, connecting the left- and right-hand walls, and connecting the top and bottom walls. This leads to CTs that are straight lines (although the presence of noise in the movement direction updates leads to some variability) that wrap around the environment (Fig. 5A, right). We find that the RNN model can learn to perform with high accuracy on CTs (median distance to end location of target-agent – RNN = 0.04 m), demonstrating the ability to learn to pursue in the more complex environment.

F.2 Rodent experiments

Behavioral apparatus and tracking. Rodent pursuit behavior was examined in a square open-field arena (62 × 62 cm) designed to enable the animal to chase a moving visual target. The arena floor consisted of transparent glass, allowing rear-projection of a visual stimulus from a projector positioned beneath the apparatus. The stimulus was a green circular target (diameter, 2.5 cm) displayed on the arena floor. A start box (28 × 19 cm) was attached to one side of the arena and connected to the main arena via a manually operated sliding door. A water spout located inside the start box delivered liquid reward (10 μL) following successful trials via a Bpod behavioral control system

bioRxiv preprint doi: https://doi.org/10.64898/2026.04.23.720457; this version posted April 27, 2026. The copyright holder for this preprint (which was not certified by peer review) is the author/funder, who has granted bioRxiv a license to display the preprint in perpetuity. It is made available under aCC-BY 4.0 International license.

running custom MATLAB scripts. To minimize uncontrolled visual landmarks and reduce spatial bias, the arena was surrounded by black-and-white curtains.

Animal behavior was recorded using an overhead camera at 30 Hz. Mouse and target positions were tracked in real time using a trained DeepLabCut model [77], enabling online detection of target interception defined by the proximity between the mouse's nose and the target center (< 1 cm). Upon interception, the target immediately disappeared, and reward delivery was triggered once the mouse returned to the start box. Target presentation, behavioral tracking signals, reward delivery, and event synchronization were coordinated through Bonsai.

Target trajectory generation. Target trajectories were generated offline using a custom simulation interface and implemented in two motion regimes: random trajectories (RT) and characteristic trajectories with periodic boundaries (CT-PB). In RT trials, the target followed pseudo-random two-dimensional trajectories with smooth directional transitions while avoiding arena boundaries. Target speed varied between 5 and 30 cm/s and trajectory duration ranged from 7 to 15 s. Each trajectory was generated uniquely across trials to prevent repetition. In CT-PB trials, the target moved at a constant speed (25 cm/s) along a straight trajectory. When the target reached an arena boundary, its position was wrapped to the opposite side while preserving speed and direction, implementing a periodic boundary condition that produced highly stereotyped trajectories across repetitions. CT-PB trials terminated after 1 min or after five full trajectory iterations.

Task structure and training. Each trial began with the mouse positioned inside the start box. At trial onset, the target appeared in the arena and the sliding door was opened manually. Target motion along the predefined trajectory was initiated once the mouse's head entered the arena. A trial was considered successful when the mouse intercepted the target; otherwise, the trial terminated without reward when the trajectory ended. Each session consisted of 30 trials, and animals typically performed one RT session and one to two CT-PB sessions per day. Mice were initially trained on RT trials until stable performance was achieved ($\geq 80\%$ interception success), after which CT-PB sessions were introduced to test whether animals could learn and exploit the repeating trajectory structure. Throughout training, animals were maintained under controlled water access ($\tilde{8}0\%$ baseline body weight) in accordance with institutional animal care guidelines.

G Decoding Analysis

To perform our decoding analyses, we focus on fully trained low-rank RNNs on the two types of pursuit tasks, i.e., when the loss function focuses on the final distance (Figs. 7, S7, and S8) and on the trial-averaged distance (Fig. S9). For a given RNN and trial type (random vs characteristic), we generated independent trials and collected the RNN-agent ($\mathbf{z}_{\text{RNN}}(t)$) and target-agent ($\mathbf{z}_{\text{target}}(t)$) trajectories. From these, we also computed the egocentric target distance as $\mathbf{z}_{\text{ego}}(t) = \mathbf{z}_{\text{target}}(t) - \mathbf{z}_{\text{RNN}}(t)$. Conjunctively, we also noted the neural activations ($r(t)$) during these trials. We refer to these targets collectively as $\mathbf{o}(t)$ below and specify whenever necessary.

Decoding setup. We trained the decoders using $\mathbf{r}(t)$ as inputs and $\mathbf{o}(t + \Delta)$ as outputs, where Δ is the temporal shift used to assess whether neural activations contain information about past states or predictions about future locations. The decoders are always trained on random trajectories and evaluated on either random or characteristic trajectories, with 320 training trials and 80 test trials. We perform 3 such train-test splits for each given data point, and save two metrics: i) the median Euclidean distance (in meters) between predicted and true positions, computed across all test samples (flattened time points and trials), and ii) the R^2 computed by first flattening x,y coordinates and then summing squared residuals over all test samples. The plots in Fig. 7 report the median, first, and third quartiles for a given rank K over both all random seeds used to train independent RNNs and the random splits. We compute the shuffled baselines by shuffling the locations within individual trials.

Architecture and training. We trained linear decoders using a ridge regression. The regularization hyperparameter α was selected via 3-fold cross-validation over the 320 training trials, evaluated on a logarithmic grid of 5 values spanning $[10^{-3}, 10^{0}]$. The value of α minimizing the median Euclidean error on the validation fold was then used to refit the decoder on all 320 training trials, and this final decoder was evaluated on the 80 held-out test trials.

bioRxiv preprint doi: https://doi.org/10.64898/2026.04.23.720457; this version posted April 27, 2026. The copyright holder for this preprint (which was not certified by peer review) is the author/funder, who has granted bioRxiv a license to display the preprint in perpetuity. It is made available under aCC-BY 4.0 International license.

886 # Supplemental Figures

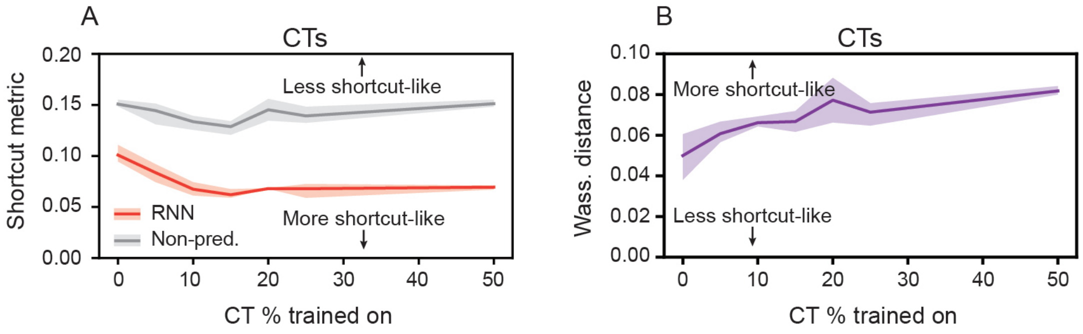

Figure S1: **Predictive behavior of RNNs on CT trials requires with exposure to CT trials during training.** (A) Shortcut metric for RNN and non-predictive models on CTs, as a function of the percent of training trials that were CTs. (B) Wasserstein distance between the distribution of RNN and non-predictive model shortcut metrics, as a function of the percent of training trials that are CTs. (A)–(B) Solid line is mean and shaded area is minimum and maximum of five independently trained networks.

bioRxiv preprint doi: https://doi.org/10.64898/2026.04.23.720457; this version posted April 27, 2026. The copyright holder for this preprint (which was not certified by peer review) is the author/funder, who has granted bioRxiv a license to display the preprint in perpetuity. It is made available under aCC-BY 4.0 International license.

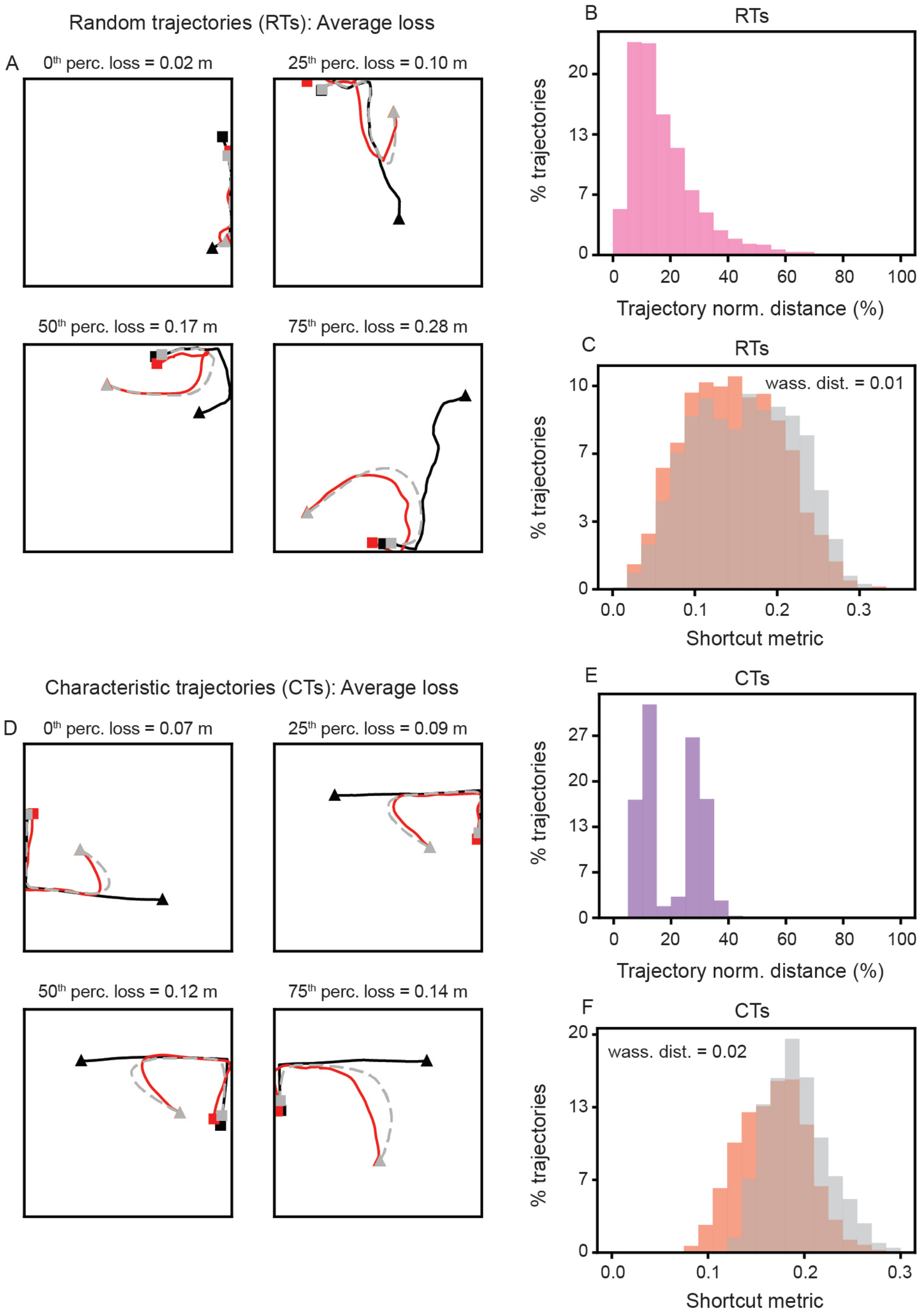

Figure S2: **Predictivity is diminished in RNN model trained to minimize average distance to target.** (A) Example RT trials for an RNN model trained to minimize the average distance to the target. Trajectories were chosen as those closest to the 0th, 25th, 50th, ad 75th percentiles of the loss distribution, respectively. (B) Distribution of normalized distance between RNN trajectory and non-predictive model on RTs. (C) Distribution of the shortcut metric for RNN and non-predictive models on RTs. Distribution was computed across 1000 trajectories for three independently trained networks. (D)–(F) Same as (A)–(C), but for CTs. Distribution are computed across 1000 trajectories for three independently trained networks.

bioRxiv preprint doi: https://doi.org/10.64898/2026.04.23.720457; this version posted April 27, 2026. The copyright holder for this preprint (which was not certified by peer review) is the author/funder, who has granted bioRxiv a license to display the preprint in perpetuity. It is made available under aCC-BY 4.0 International license.

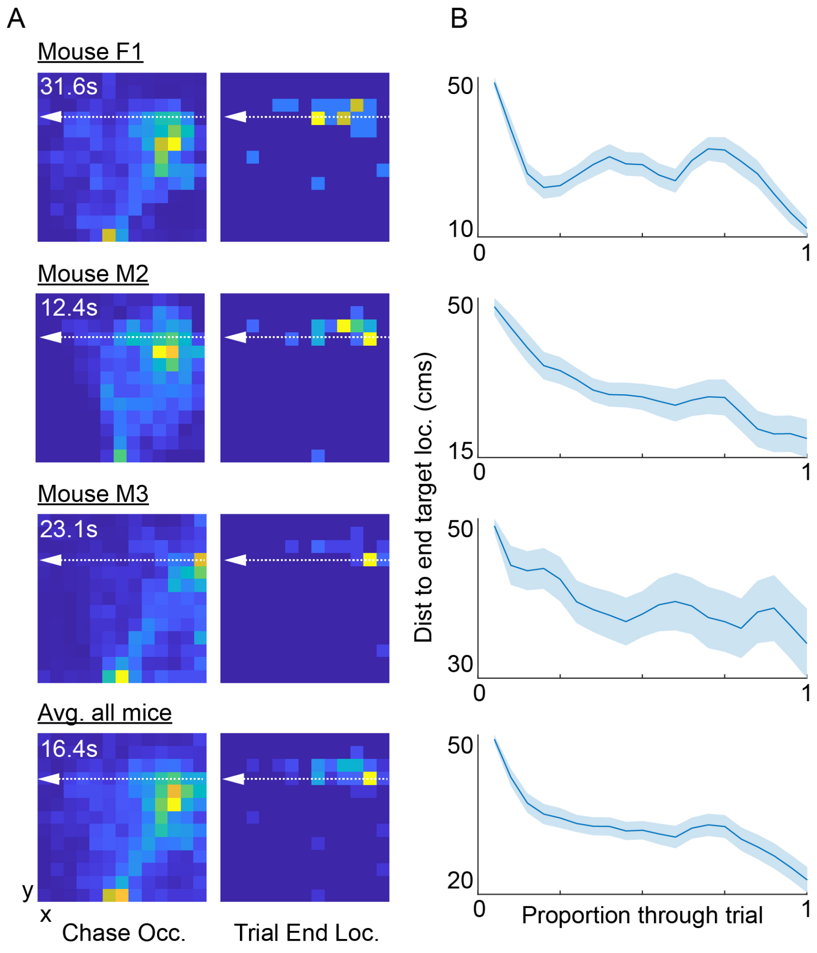

Figure S3: **Mice learn to wait during pursuit in an environment with periodic boundaries.** (A) Left column, occupancy map showing time in each arena position for 3 mice (F1, M2, M3) during periodic boundary pursuit trials. Bottom row depicts averages over all subjects. Color axis depicts occupancy in seconds (blue to yellow, zero to maximum occupancy). White numeric values in upper left corners indicate time spent in most occupied locations. Right column, occupancy map showing time in each arena position at the end of the chasing trial (including both successful and failure trials). White dashed arrow depicts trajectory of target. (B) As in Figure 5E, the average distance between the current location of the mouse vs the location of the target the end of the trial. Mice are ordered as in (A). All mice exhibited "waiting" strategies in their behavior as demonstrated by a plateau in the distance metric through the middle of the trial. Bottom row depicts average over all mice.

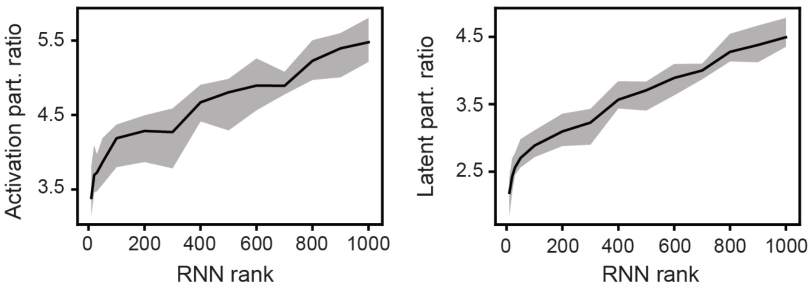

Figure S4: **High-dimensionality of RNN activity and latent variables is also observed when using participation ratio, instead of PCA, to define dimensionality.** Same as Fig. 6B, but for participation ratio instead of PCA.

bioRxiv preprint doi: https://doi.org/10.64898/2026.04.23.720457; this version posted April 27, 2026. The copyright holder for this preprint (which was not certified by peer review) is the author/funder, who has granted bioRxiv a license to display the preprint in perpetuity. It is made available under aCC-BY 4.0 International license.

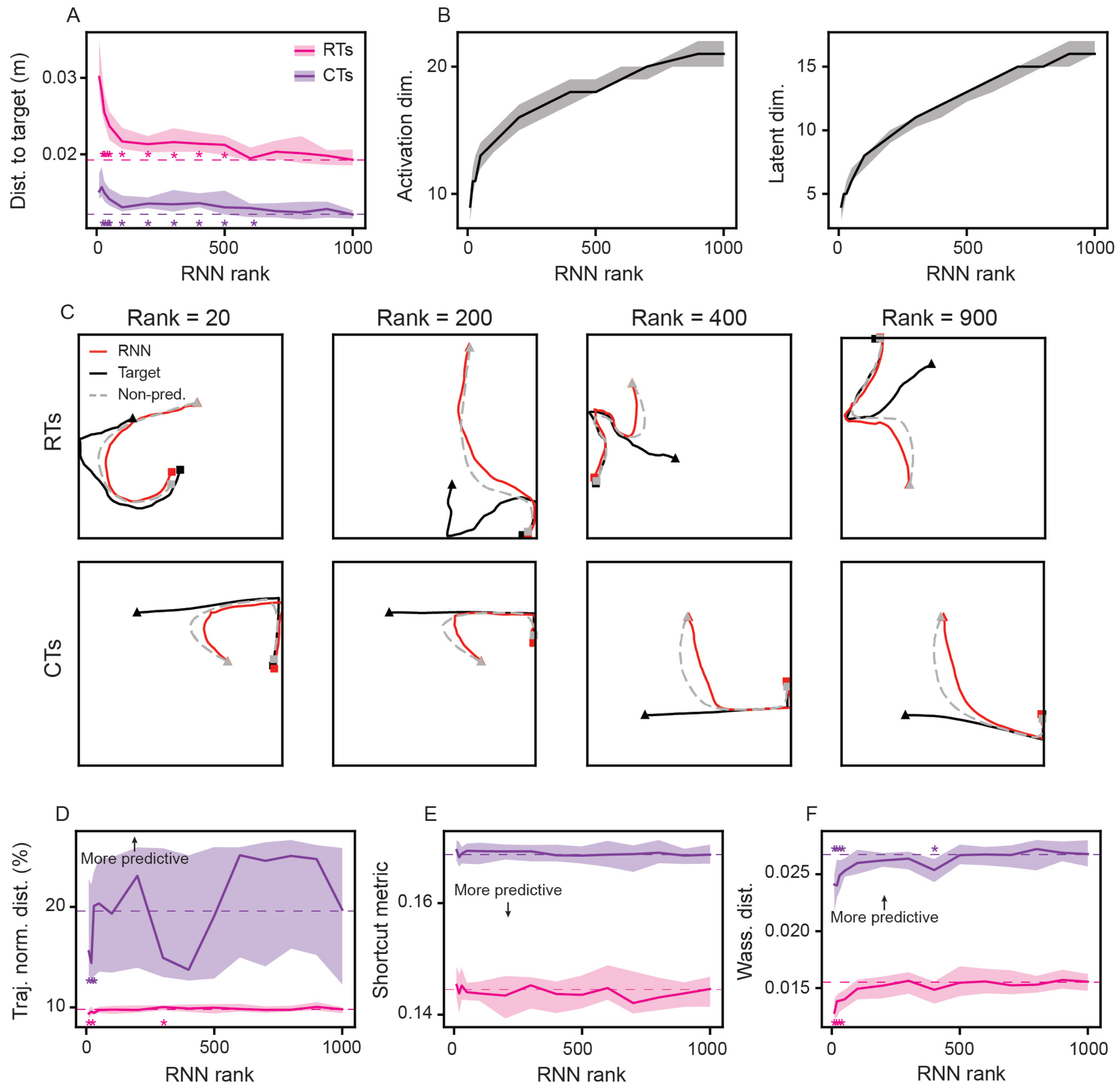

Figure S5: **Predictivity does not emerge with increased RNN model rank, when training to minimize average distance to target.** Same as Fig. 6, but for RNN whose loss function is the average distance between the target-agent and the RNN-agent, instead of the distance at the final time-point in the trial.

bioRxiv preprint doi: https://doi.org/10.64898/2026.04.23.720457; this version posted April 27, 2026. The copyright holder for this preprint (which was not certified by peer review) is the author/funder, who has granted bioRxiv a license to display the preprint in perpetuity. It is made available under aCC-BY 4.0 International license.

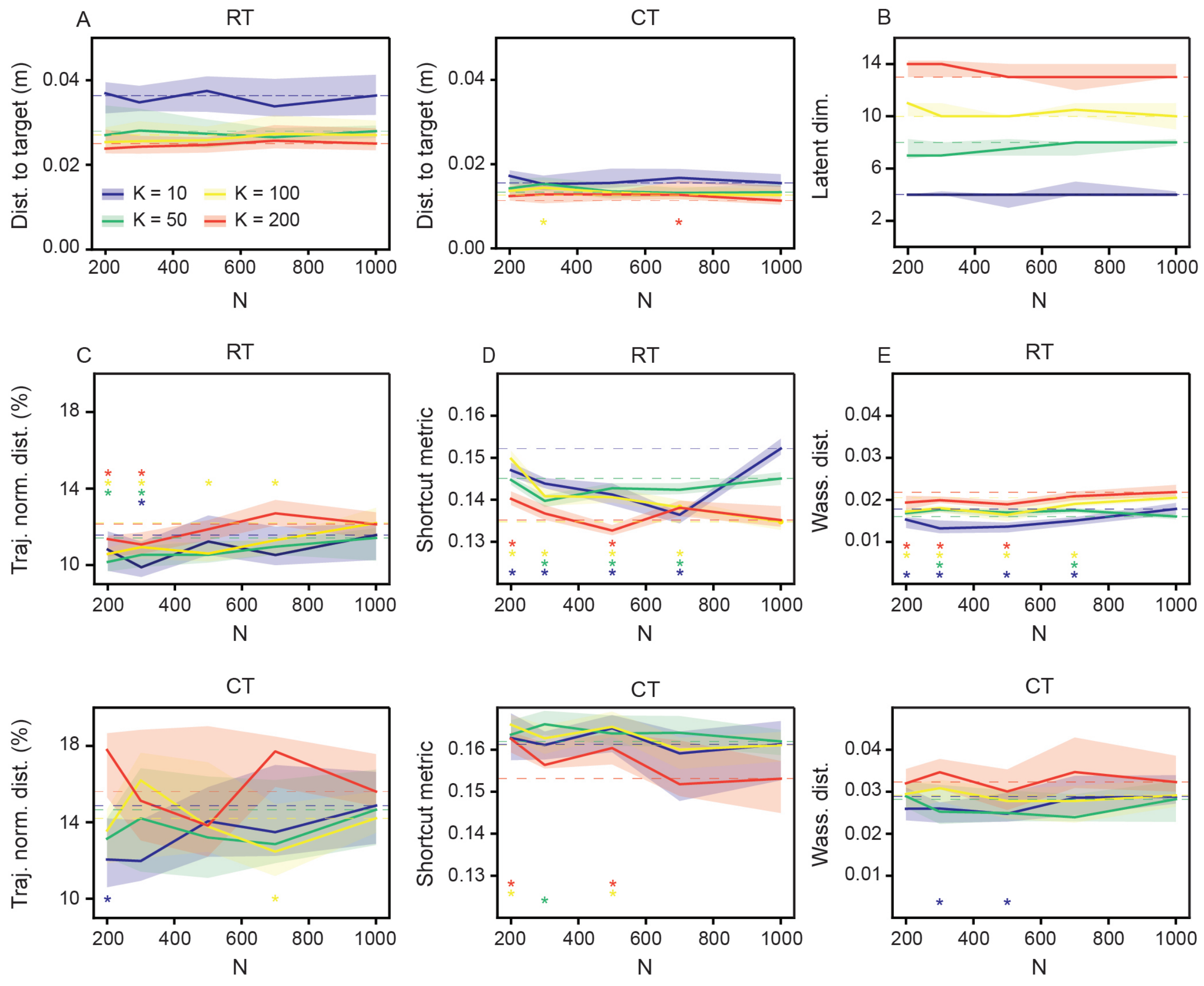

Figure S6: **Predictivity of low rank RNNs depends on rank not network size.** (A) Distance to end location of target-agent for RNNs of fixed rank and varying number of hidden units (N). (B) Dimensionality (as estimated by PCA) of latent variables, as a function of N. (C) Normalized distance between RNN and non-predictive control model trajectories, as a function of N for RTs (top row) and CTs (bottom row). (D) Shortcut metric, as a function of N. (E) Wasserstein distance between RNN and non-predictive model shortcut metric distrubtions, as a function of N. (A)–(E) Solid line is median and shaded area is $\pm$ standard deviation across 20 independently trained networks. Stars denote N values where two-sample Kolmogorov test showed significance with respect to $N = 1000$ model distribution ($p-$value < 0.05; dashed lines denote $N = 1000$ model median values).

bioRxiv preprint doi: https://doi.org/10.64898/2026.04.23.720457; this version posted April 27, 2026. The copyright holder for this preprint (which was not certified by peer review) is the author/funder, who has granted bioRxiv a license to display the preprint in perpetuity. It is made available under aCC-BY 4.0 International license.

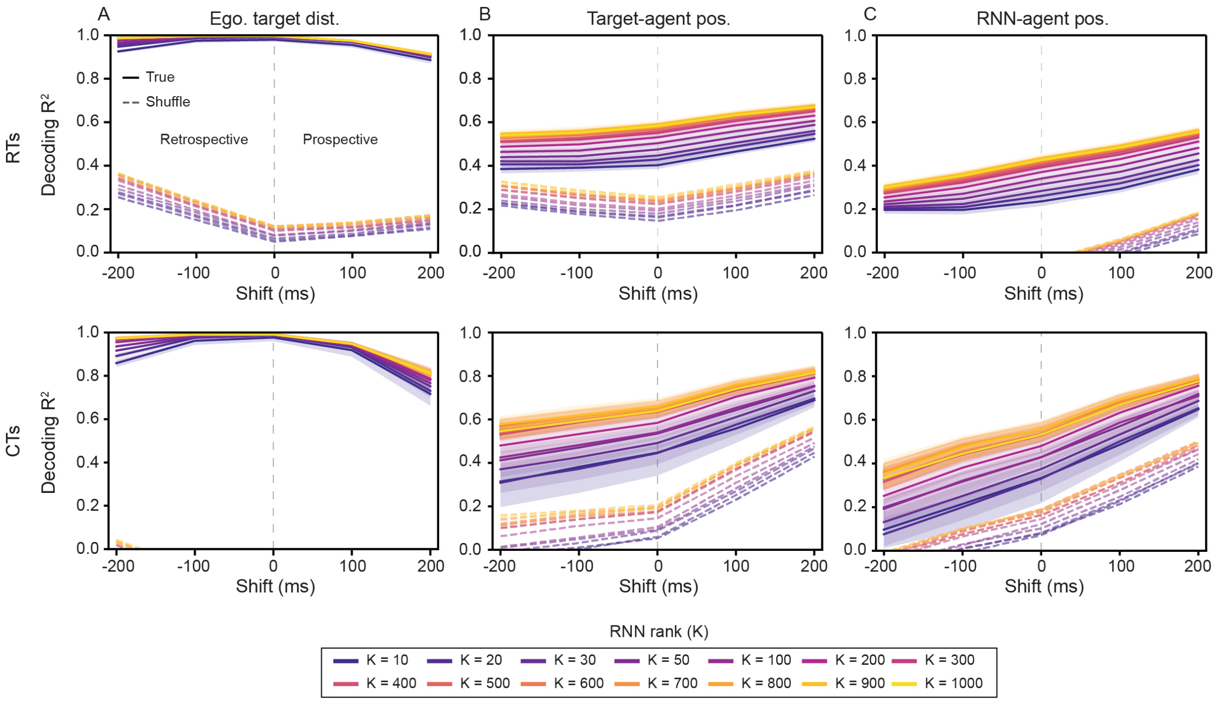

Figure S7: **Decoding R^2 of RNN-agent and target-agent position increases with increasing RNN model rank.** Same as Fig. 7, but with decoding R^2 instead of decoding error.

bioRxiv preprint doi: https://doi.org/10.64898/2026.04.23.720457; this version posted April 27, 2026. The copyright holder for this preprint (which was not certified by peer review) is the author/funder, who has granted bioRxiv a license to display the preprint in perpetuity. It is made available under aCC-BY 4.0 International license.

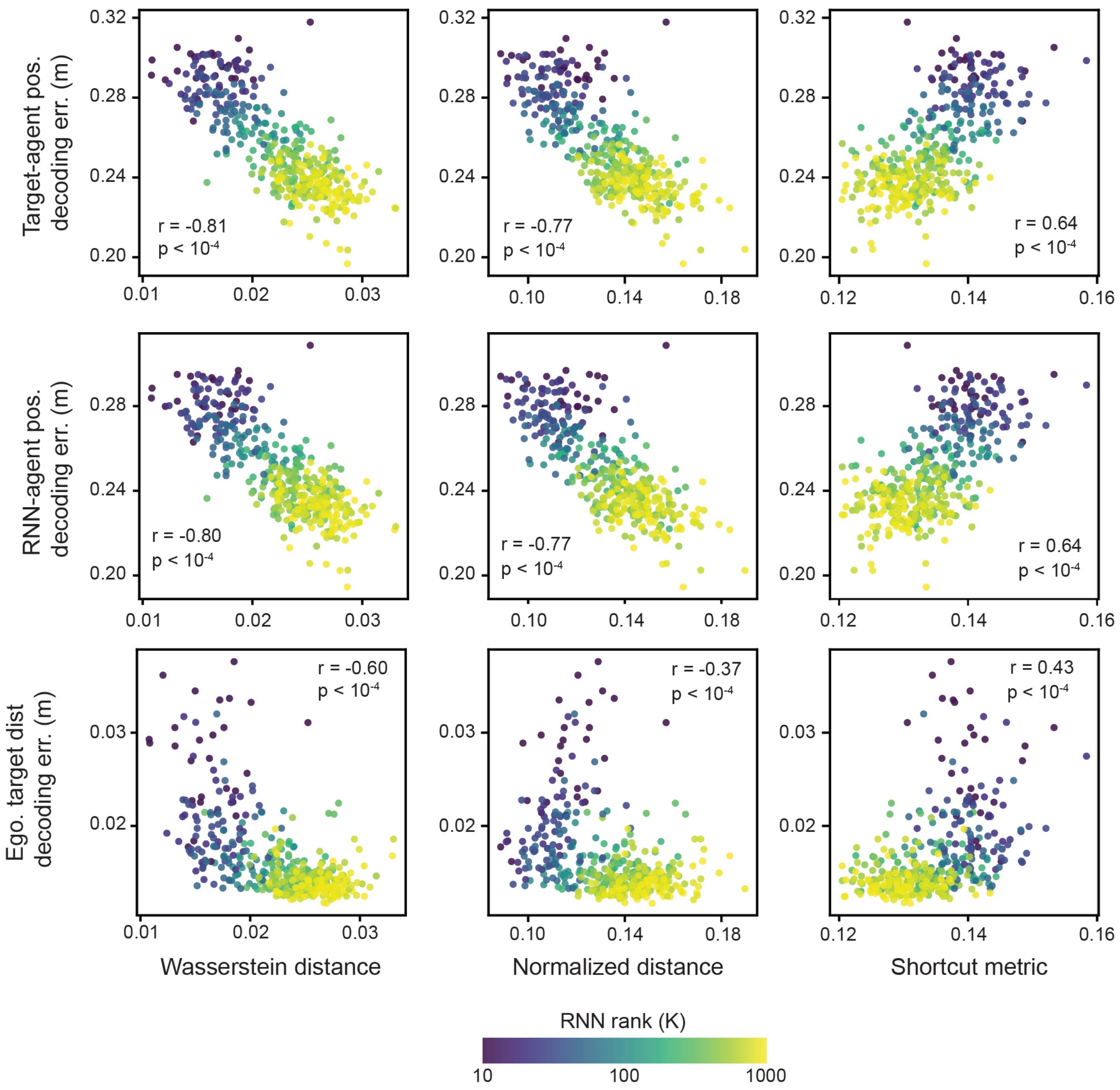

Figure S8: **Strong correlation between predictive behavior and linear decoding error of allocentric information in RNNs.** (Top row) Target-agent position decoding error as a function of Wasserstein distance, trajectory normalized distance, and shortcut metric, for all trained networks that achieved final distance to target-agent < 0.10 m ($n = 415$). (Middle row) RNN-agent position decoding error as a function of Wasserstein distance, trajectory normalized distance, and shortcut metric. (Bottom row) Egocentric target distance decoding error as a function of Wasserstein distance, trajectory normalized distance, and shortcut metric. Linear regression r and $p-$values reported in each subplot. Color of each dot denotes rank of corresponding RNN.

bioRxiv preprint doi: https://doi.org/10.64898/2026.04.23.720457; this version posted April 27, 2026. The copyright holder for this preprint (which was not certified by peer review) is the author/funder, who has granted bioRxiv a license to display the preprint in perpetuity. It is made available under aCC-BY 4.0 International license.

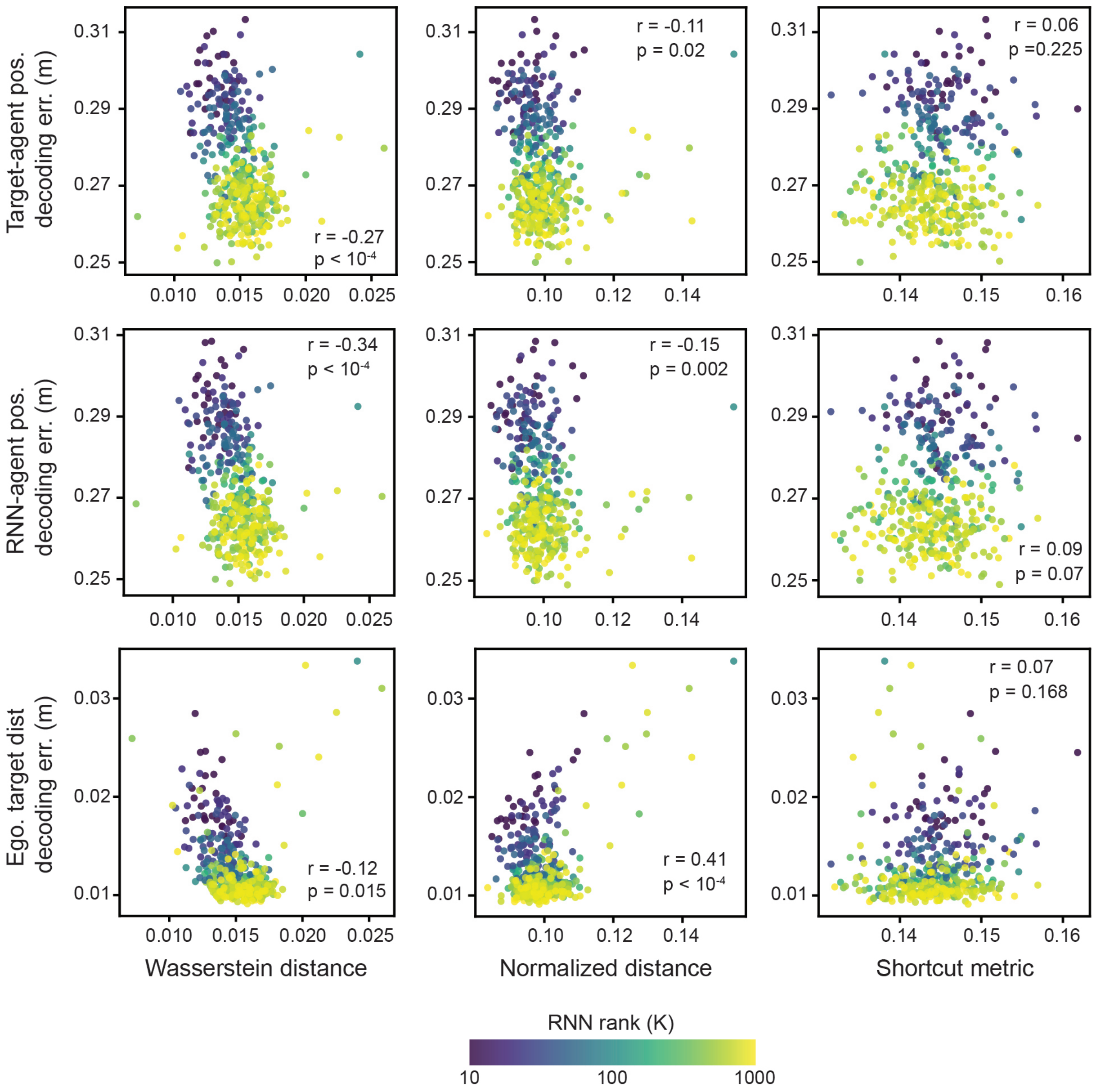

Figure S9: **Weak correlation between predictive behavior and linear decoding error of allocentric and egocentric information in RNNs trained to minimize average distance.** Same as Fig. S8, but for RNN whose loss function is the average distance between the target-agent and the RNN-agent, instead of the distance at the final time-point in the trial. $n = 411$ networks are plotted.
